# Associations between combined overweight and obesity, lifestyle behavioural risk and quality of life among Australian regional school children: baseline findings of the Goulburn Valley health behaviours monitoring study

**DOI:** 10.1186/s12955-019-1086-0

**Published:** 2019-01-18

**Authors:** Erin Hoare, Nicholas Crooks, Joshua Hayward, Steven Allender, Claudia Strugnell

**Affiliations:** 1 Food & Mood Centre, IMPACT SRC, School of Medicine, Deakin University, 1 Gheringhap Street, Geelong, Australia; 2 Global Obesity Centre, Centre for Population Health Research, Deakin University, Geelong, Australia

**Keywords:** Psycho-social health, Obesity prevention, Diet, Physical activity, School children

## Abstract

**Introduction:**

Health related quality of life is a multi-dimensional construct of particular interest in determining the consequences of illness and disease. This study aimed to determine the relationships between overweight/obesity, and associated obesogenic risk behaviours with health related quality of life and physical, social, emotional and school sub-domains, among a large cohort of Australian primary school children.

**Methods:**

The data were derived from the Goulburn Valley Health Behaviours Monitoring study whereby a census-styled school recruitment process and high participatory opt-out (passive) procedure was employed. All primary schools in three Local Government Areas were invited to participate between July-September 2016 with 39/62 (62%) of schools participating and 1606/2034 (79%) students in Grade 2 (aged approx. 7-8 years), Grade 4 (aged approx. 9-10 years) and Grade 6 (aged approx. 11-12 years) participating. Measured height and weight were collected among participating students and older children (Grade 4 and 6) who also completed a self-report behavioural questionnaire, including the paediatric quality of life inventory.

**Results:**

Among 809 children aged 9 to 12 years, there were 219 (27.1%) classified as overweight/obese. Male children classified as overweight/obese reported significantly lower health related quality of life in the physical functioning and global functioning scores, compared to normal weight males. Significantly higher quality of life scores were observed among all children who met the physical activity recommendations on five out of the seven previous days. Significantly higher scores were observed among males adhering to the daily screen time recommendations, and among those meeting daily recommendations for fruit consumption. Among male school children, soft drink consumption was associated to lower health related quality of life.

**Conclusion:**

Although cross-sectional, these findings highlight children with overweight/obesity and some underlying lifestyle behavioural risk factors, had significantly lower healthy-related quality of life, although this was observed most consistently among male school children. These findings have not previously been identified in young children and highlights the need to consider mental and emotional health in public health efforts to prevent obesity.

**Trial Registration:**

ANZCTR Trial Registry: ACTRN12616000980437 retrospectively registered 26 July 2016

**Electronic supplementary material:**

The online version of this article (10.1186/s12955-019-1086-0) contains supplementary material, which is available to authorized users.

## Introduction

Childhood obesity is associated with a range of negative health comorbidities and future health outcomes [1]. Epidemiological evidence suggests obesity prevalence is continuing an upward trajectory [2–4]. Despite scientific advances in identifying the biological, environmental, social and other drivers of unhealthy weight gain [5, 6] public health efforts are on-going in how to best tackle the complexity of overweight/obesity, with many previous prevention attempts described to be largely unsuccessful [7].

The negative psycho-social impact of overweight/obesity is also widely recognised [8, 9]. Common mental disorders such as depression and anxiety are known to co-occur with overweight/obesity, and young people and adults reporting unhealthy weight status are also likely to report poorer individual, familial and community functioning [6, 10, 11]. There are known physiological links between behaviours underpinning obesity such as diet, physical inactivity and psycho-social health, these relationships are therefore assumed to be bi-directional [12]. These relationships are of critical importance during childhood, as it is during this development life period that lifestyle habits are formed, and overweight/obesity and psychosocial-related problems often first occur that track into adolescence and adulthood [13, 14].

Health-related quality of life (HRQoL) is of particular interest among children because it is a comprehensive and multi-dimensional construct that incorporates physical, emotional, social and school functioning [15]. HRQoL broadly refers to the extent to which an individuals can successfully function in daily life, and perceived wellness across physical, emotion and social domains [15]. Among young people, functioning is also extended to include a school domain given the large proportion of time young people spend attending school [16]. These domains are of great interest in the study of obesity and underpinning risk factors given comorbidity and the known negative impact of unhealthy weight on daily functioning and psychosocial health. There is also evidence to suggest learning outcomes are influenced by young children’s health and lifestyle behaviours. A large Australian population study found smaller hippocampal volume among individuals who consumed typically unhealthy diets over time, compared to those who reported traditional healthful diets [17]. Highlighting, that it is possible that daily lifestyle behaviours have implications for brain health and subsequent learning and memory outcomes.

Overweight/obesity among young people has previously been shown to be associated with poor physical quality of life, but unrelated to emotional, school or social functioning [18]. Patterns appear to differ by age group with more recent data showing poorer physical and psychosocial functioning increasingly associated with BMI among older aged adolescents, but not younger [19]. These population-cohort study designs are now more than a decade old and today’s children live in very different environment to those studied in these earlier studies. As one example, rapid increases in the availability and use of smart technologies has led to differences in the way modern young people socialise, seek information, and communicate [20]. In addition, experiences of weight, psycho-social health and lifestyle behaviours appear to be related to area of residence, with regional and rural young people experiencing unique risks for health outcomes [21]. There is a need for epidemiological evidence to incorporate modern experiences of young people’s daily behaviours and the context in which such behaviours occur.

Furthermore, sex and age-specific associations appear to exist. Associations have been observed between HRQoL and overweight/obesity among females adolescents [22], and to varying levels among adults [23], and among clinical populations [24]. A study in 2014 examined HRQoL and health behaviours among Australian school children but considered global HRQoL only, as opposed to individual sub-domains [25]. This study also failed to examine sex-differences which are known to occur in lifestyle health behavioural risks during younger years. Similar limitations were observed in a recent international analysis of HRQoL and lifestyle behaviour clusters in school-children across 12 countries, which examined total summary HRQoL scores across total populations only [26].

Further compounding these inconsistencies in the literature is the fact that these studies employed opt-in (active) consent procedures which typically achieve student response rates between 30-60% [27]. It has been demonstrated that opt-in approaches influences estimates of overweight and obesity when compared to high participatory opt-out (passive) approaches (typically achieving student response rates ≥ 80%) [28]. Subsequently, students recruited under opt-in procedures are typically healthier, with significant underestimation of overweight/obesity among girls as high as -4.6 percentage points; and this underestimation is likely to influence observed relationships between HRQoL and overweight/obesity.

Therefore, the aims of this study was to examine the relationship between global health related quality of life and sub domains (physical, social, emotional and school functioning) and overweight/obesity in a high participatory opt-out study of Australian school children living in regional and rural Victoria. This study also aimed to investigate the relationship between key underlying obesogenic risk factors, fruit and vegetable consumption, soft drink consumption, physical inactivity and screen time.

## Methods

### Study design, school and student recruitment

This study used an identical recruitment procedure and methodology of Great South Coast childhood obesity monitoring system in South-West Victoria, Australia which have been reported in detail elsewhere [29]. Briefly, all primary schools within three [3] local government authorities (LGAs) were invited and participated in Term 3 (July – September) 2016. All participating primary schools (government, independent and catholic) (39/62) used an opt-out (passive) approach to invite students in Grade 2, Grade 4 and Grade 6 [1606/2034 participate (79%)].

### Measures

Participating students were invited to have their height and weight measured by trained data collectors during class time (all Grades) and complete a self-report demographic (date of birth, gender, residential postcode, language spoken mostly at home and ancestry) and health-behaviours (physical activity, sedentary behaviour [30, 31], dietary [32], sleep and perceived quality of life) [33] questionnaire using an electronic tablet (Grade 4 and Grade 6 only).

Health related quality of life, the primary outcome of interest, was measured using the Pediatric Quality of Life Inventory (PedsQoL) which asked participants to report over the previous month how much of a problem various items had been for them [34]. The PedsQoL comprises 23 items relating to physical functioning (8 items, e.g., ‘*it is hard for me to walk more than one block*’), emotional functioning (5 items, e.g., ‘*I feel sad or blue’*), social functioning (5 items, e.g., ‘*I have trouble getting along with other kids’*) and school functioning (5 items, e.g., ‘*it is hard to pay attention in class’*). Responds are scored *never* (0), *almost never* [1], *sometimes* [2], *often* [3], or *almost always* [4]. Items were reversed scored and transformed to a 0-100 (0=100, 1=75, 2=50, 3=25, 4=0) scale so that higher scores reflected better HRQoL, as per scoring guidelines. Scales scores were computed from sum and averaged total items within each scale, and a total score (Global) was computed from the sum and average of all items across all four scales. The PedsQoL has been shown to be a reliable and valid measure of health related quality of life among children [33–35].

Height was measured to the nearest 0.1 centimetre (cm) using a portable stadiometer (Charder HM-200P Portstad, Charder Electronic Co Ltd, Taichung City, Taiwan) and weight to the nearest 0.01 kilogram (kg) using an electronic weight scale (A&D Precision Scale UC-321; A7D Medical, San Jose, CA). Two measurements were taken unless the first two measures differed by at-least 0.5 cm or 0.1 kg, in which case a third measure was required. Students wore light clothing and no shoes (e.g. removed jumpers/blazers, items in pockets). All data were typically collected during one class period, approximately 50 minutes during school-time.

Students adherence to the national physical activity and sedentary guidelines [36] was measured using select items from the Core Indicators and Measures of Youth Health – Physical Activity & Sedentary Behaviour Module [30] and the School Health Action, Planning and Evaluation System (SHAPES) Physical Activity Questionnaire [31]. Dietary data was gathered using a modified version of the Simple Dietary Questionnaire measuring the frequency of consumption of fruit and vegetables, take-away foods, packaged snacks, water and sweetened beverages [32]. Componented of students sleep, including frequency, duration and quality were measured using modified items from the Adolescent Sleep Health Survey (ASHS) [37], the School Sleep Hygiene Survey (SSHS) [38], the Children’s Sleep Health Questionnaire (CSHQ) [39], and the Pittsburgh Sleep Quality Index (PSQI) [40].

### Data management

HRQoL summary scores were calculated using the responses in the 4 domains of the health-related quality of life inventory and the continuous summary scores used in analysis [34]. Children’s weight status was calculated using the means of the 2, or 3 if required, measures of height and weight. Age and sex-specific body mass index (BMI) z-scores were determined and children’s weight status categorised using the International Obesity Task Force cut-points for comparability with previous studies [41]. Behavioural data were recoded as binary variables (Yes/No) for the proportion of respondents meeting the Australian national physical activity and sedentary behaviour guidelines for children in the last 5 or more days (5-12 years) [36]; proportion meeting the Australian Dietary guidelines for fruit and vegetable intake [42]. Proportions for usually consuming takeaway meals once a week or less, usually consuming sugar-sweetened beverages less than once/day (soft drinks, cordial, fruit or sports drinks) and usually consuming ≥5 cups/day of water were also calculated. Individuals Relative Socio-economic Advantage and Disadvantage (IRSAD) was derived from the Australian Bureau of Statistics (ABS) Socio-Economic Indexes for Areas (SEIFA) index from the 2011 Australian Census [43] and participants categorised into quintiles based on self-reported postcode and/or suburb. A measure of cultural and linguistic diversity was determined based on language predominantly spoken at home which was dichotomised into English speaking and Language other than English.

### Ethics

This study received ethical approvals from Deakin University’s Human Research Ethics Committee (2014_289), the Victorian Department of Education and Training (2015_002622) and the Catholic Archdiocese of Sandhurst. Principals of Independent schools gave approval at the school-level and an opt-out process was approved and utilised in this study. All students of approving schools were provided with a plain language statement and opt-out form after a presentation to school children from the research team in class-time. Participants were only required to return a signed opt-out form if they or their parents/guardians did not wish for their child to participate. Children who did not wish to be measured or surveyed did not have to participate, regardless of whether or not they had a signed opt-out form.

### Statistical analysis

All analysis were conducted in Stata/SE Corp Version 15.0. Significance was assumed at p<0.05 however the Bonferroni corrected equivalent significance cut-off was p<0.01 (0.05/5 independent predictor variables). Proportions were calculated and significance in differences between overweight/obesity was tested using logistic regression models. Linear regression models were used to assess the relationship between each HRQoL domain as the dependent variable, and each lifestyle behavioural risk entered into separate models. Coefficients are reported as unstandardized beta coefficients and 95% confidence intervals. Age in whole years was included as a covariate and models were adjusted for potential clustering effect of school in which the participant attended. Linear regression models were stratified by sex. Bonferroni corrections for multiple comparison were used.

## Results

Of the 62 schools invited to participate in the Goulburn Valley Health Behaviours Monitoring Study (GVHBMS), 39 schools (62%) consented and were subsequently included in the baseline (2016) data collection wave. Data collection included children in Year 2, 4 and 6 however children in Year 2 did not complete the self-report behavioural questionnaire component and were not included in this study (n=561). Overweight/obesity prevalence among the participating sample was just over a quarter (27.1%) and this slightly greater among children aged 9-10 years (27.5%) compared to children aged 11-12 years (26.6%) although non-significant (Table 1, all IOTF). Approximately half the participating sample were female (52.5%) and a slightly greater proportion of Year 4 students (55.8%) compared to Year 6 (44.3%) were represented in the final sample. Overweight/obesity was similar across gender and year level. The majority of children spoke English at home (85.3%) though overweight/obesity was greater among non-English speakers (34.2%) compared to English speakers (26.1%). No significant differences were observed in overweight/obesity prevalence relative to the sociodemographic level of disadvantage of participating children.

**Table 1:** Participant demographic characteristics by overweight/obesity status

	All^1^	Healthy Weight	Overweight/obese (IOTF)	*P* value^2^
Participants (%)	809 (100)	590 (72.9)	219 (27.1)	
Age n (%)				NS
9-10 years	444 (54.9)	322 (72.5)	122 (27.5)	
11-12 years^3^	365 (45.1)	268 (73.4)	97 (26.6)	
Sex				NS
Female n (%)	384 (52.5)	275 (71.6)	109 (28.4)	
Male n (%)	425 (47.5)	315 (74.1)	110 (25.9)	
BMI z-score m(SD)	0.67 (1.30)	0.12 (0.79)	2.18 (1.21)	NS
School year n (%)				NS
Year 4	451 (55.8)	330 (73.2)	121 (26.8)	
Year 6	358 (44.3)	260 (72.6)	98 (27.4)	
Language at home n (%)				NS
English	678 (85.3)	501 (73.9)	177 (26.1)	
Other	117 (14.7)	77 (65.8)	40 (34.2)	
SEIFA n (%)				NS
Quintile 1 (Lowest)	384 (47.5)	269 (70.1)	115 (30.0)	
Quintile 2	160 (19.8)	Suppressed	Supressed	
Quintile 3	28 (3.5)	Supressed	Supressed	
Quintile 4	237 (29.3)	180 (76.0)	57 (24.1)	

^1^Percentages relate to proportion of sample overall. ^2^P-value relates to significant difference between normal weight and overweight/obesity. ^3^Three children were aged 13 and were included in 11-12 year age group. Supressed = values not shown due to very small participant numbers.

Some health behavioural and HRQoL differences were observed between children classified as overweight/obese and healthy weight children (Table 2). Soft drink non-consumers had the lowest rates of overweight/obesity (24.7%), and this was significantly different to children who consumed such drinks every day (35.6%). Of children classified as healthy weight, 40.5% were sufficiently active, compared to 27.4% of overweight /obese children. Individuals who were classified as overweight/obese scored significantly lower on all domains of HRQoL and the total summary score, compared to healthy weight children (Table 2).

**Table 2:** Health behavioural characteristics and Health Related Quality of Life scores by overweight/obesity status

	All^1^	Healthy Weight	Overweight/obese (IOTF)	*P* value^2^
Total	809 (100)	590 (72.9)	219 (27.1)	
Fruit				NS
< 2 serves per day n (%)	211 (26.1)	146 (24.8)	65 (29.7)	
Fruit >=2 or more serves per day n (%)	598 (73.9)	444 (75.3)	154 (70.3)	
Vegetable				NS
< 5 serves per day	691 (85.4)	499 (84.6)	192 (87.7)	
>=5 or more serves per day n (%)	118 (14.6)	91 (15.4)	27 (12.3)	
Soft-drink consumption				
Rarely or never	241 (29.8)	187 (31.7)	54 (24.7)	*p*=0.011^3^
Every second day to once a fortnight	330 (40.8)	243 (41.2)	87 (39.7)	
Almost every day or more	238 (29.4)	160 (27.1)	78 (35.6)	
Takeaway consumption				
Once a fortnight or less	469 (58.0)	356 (60.3)	113 (51.6)	*p*=0.036^4^
Once a week	237 (29.3)	164 (27.8)	73 (33.3)	
2-4 times or more	63 (7.8)	40 (6.8)	23 (10.5)	
Everyday	40 (5.0)	30 (5.1)	10 (4.6)	
Water recommendation				NS
<5 glasses a day	352 (43.5)	256 (43.4)	96 (43.8)	
5-8 glasses a day	322 (39.8)	229 (38.8)	93 (42.5)	
≥8 glasses day	135 (16.7)	105 (17.8)	30 (13.7)	
Physical activity				*p*=0.001
Not met on 5 out of 7 days	510 (63.0)	351 (59.5)	159 (72.6)	
Met physical activity on previous 5 out of 7 days n (%)	299 (37.0)	239 (40.5)	60 (27.4)	
Screen time				NS
Not met on 5 out of 7 days	190 (23.5)	133 (22.5)	57 (26.0)	
Met screen time on 5 of previous 7 days n (%)	619 (76.5)	457 (77.5)	162 (74.0)	
Health Related Quality of Life m(95%CI) scores out of maximum possible 100				
Physical	84.1 (83.1, 85.1)	85.2 (84.1, 86.3)	81.1 (78.9, 83.4)	*p*=0.000
Emotional	71.2 (69.8, 72.6)	72.1 (70.6, 73.7)	68.7 (65.8, 71.7)	*p*=0.031
Social	78.4 (77.0, 80.0)	79.9 (78.4, 81.5)	74.4 (71.3, 77.6)	*p*=0.001
School	75.0 (73.7, 76.2)	75.7 (74.3, 77.1)	73.0 (70.5, 75.6)	*p*=0.060
Global	78.1 (77.1, 79.1)	79.2 (78.1, 80.3)	75.2 (72.9, 77.4)	*p*=0.001

^1^Percentages relate to proportion of sample overall. ^2^*P*-value relates to significant difference between normal weight and overweight/obesity. ^3^Overwieght/obesity prevalence was significantly different to those who consumed soft drinks every days. ^4^Overwieght/obesity prevalence was significantly different to those who consumed takeaway foods 2-4 times per week

Females scored significantly lower on emotional functioning compared to males, and higher on school functioning scale (Additional file 1: Table S1). Among males, overweight/obesity was significantly associated to physical and global domains of HRQoL, and these relationships were independent of age and any potential clustering effect of schools (Table 3).

**Table 3:** Linear regression models for relationship between health related quality of life (DV, scores possible from 20-100 where higher scores reflect higher health related quality of life) and overweight/obesity (IV, overweight/obese=1 compared to healthy weight=0) for males and females, accounting for age and clustering effects of school which participant attended

	Males			Females		
	*b*	*95%CI*	*p*	*b*	*95%CI*	*p*
Physical (0 = Healthy weight, 1 = Ov/Ob)	**-6.19**	**-10.33, -2.05**	**0.005**	-1.81	-5.63, 2.01	0.342
Emotional (0 = Healthy weight, 1 = Ov/Ob)	-4.05	-7.94, -0.17	0.041	-2.51	-6.96, 1.94	0.259
Social (0 = Healthy weight, 1 = Ov/Ob)	-5.62	-10.84, -0.39	0.036	-5.42	-10.00, -0.85	0.022
School (0 = Healthy weight, 1 = Ov/Ob)	-2.27	-6.35, 1.79	0.263	-3.40	-8.47, 1.67	0.181
Global (0 = Healthy weight, 1 = Ov/Ob)	**-4.73**	**-8.17, -1.29**	**0.009**	-3.21	-6.82, 0.41	0.080

Achieving sufficient physical activity on five out of the previous 7 days, consuming two serves of fruit or more per day, and adhering to daily screen time recommendations of no more than 2 hours per day, was significantly related to higher HRQoL scores among males (Table 4). Meeting physical activity guidelines was significantly associated to increased HRQoL scores among females. Soft drink consumption was associated to lower HRQoL scores among males only.

**Table 4:** Linear regression models for obesogenic risk behaviours (IV) and health related quality of life (DV, scores possible from 20-100 where higher scores reflect higher health related quality of life) for males and females, accounting for age and clustering effects of school which participant attended

	Males			Females		
	*b*	*95%CI*	*p*	*b*	*95%CI*	*p*
*Physical activity* ^*1(=>5+day)*^ *(0 = not met, 1= met)*						
Physical	**5.84**	**2.50, 9.18**	**0.001**	**4.58**	**1.15, 8.00**	**0.010**
Emotional	**7.14**	**3.08, 11.21**	**0.001**	**6.11**	**1.95, 10.27**	**0.005**
Social	**7.28**	**2.96, 11.59**	**0.002**	**5.43**	**2.07, 8.79**	**0.002**
School	**6.51**	**1.62, 11.40**	**0.011**	**4.77**	**1.49, 8.06**	**0.006**
Global	**6.63**	**3.27, 9.98**	**0.000**	**5.09**	**2.27, 7.90**	**0.001**
*Screen time* ^*1(>=5+day)*^ *(0 = not met, 1= met)*						
Physical	**6.89**	**3.88, 9.90**	**0.000**	4.14	0.73, 7.56	0.019
Emotional	**9.22**	**5.23, 13.22**	**0.000**	2.46	-2.63, 7.54	0.332
Social	**9.67**	**4.99, 14.35**	**0.000**	**6.38**	**2.01, 10.75**	**0.006**
School	**9.47**	**4.23, 14.71**	**0.001**	4.16	-0.16, 8.47	0.058
Global	**8.47**	**4.97, 11.97**	**0.000**	4.18	0.70, 7.66	0.020
*Fruit consumption* ^*2*^ *(0 = not met, 1= met)*						
Physical	**6.34**	**2.45, 10.23**	**0.002**	6.22	1.11, 11.32	0.019
Emotional	**5.97**	**2.84, 9.09**	**0.000**	6.91	-1.20, 15.03	0.092
Social	**5.91**	**2.04, 9.78**	**0.004**	**8.66**	**3.01, 14.31**	**0.004**
School	4.34	0.60, 8.10	0.024	5.98	0.31, 11.65	0.039
Global	**5.72**	**2.85, 8.60**	**0.000**	6.73	1.19, 12.27	0.019
*Vegetable consumption* ^*3*^ *(0 = not met, 1= met)*						
Physical	1.86	-2.57, 6.29	0.399	**4.92**	**1.23, 8.60**	**0.011**
Emotional	1.55	-3.09, 6.20	0.501	0.36	-7.44, 8.15	0.926
Social	3.41	-1.12, 7.95	0.135	0.93	-4.68, 6.54	0.739
School	4.62	-0.22, 9.46	0.061	0.16	-4.76, 5.07	0.949
Global	2.79	-0.98, 6.55	0.141	2.10	-2.30, 6.50	0.339
*Soft drink consumption* *(0 = rarely or never consumed 1=occasionally to everyday)*						
Physical	**-3.95**	**-6.88, -1.02**	**0.010**	-1.78	-4.79, 1.24	0.238
Emotional	-1.80	-5.84, 2.24	0.372	-2.50	-6.77, 1.76	0.240
Social	**-5.60**	**-9.69, -1.50**	**0.009**	-2.33	-6.14, 1.48	0.223
School	-4.72	-9.11, -0.33	0.036	-3.22	-7.22, 0.78	0.111
Global	**-4.00**	**-6.73, -1.26**	**0.005**	-2.46	-5.48, 0.56	0.107

Note: *b* is unstandardized. ^1^Meeting physical activity guidelines on previous 5 out of 7 days compared to those who did not meet guidelines for minimum 5 days. ^2^2 serves a day or more compared to less than two serves per day. ^3^Five serves a day or more compared to less than five serves a day.

## Discussion

The relationship between HRQoL and overweight obesity among regional and rural school-aged children was apparent, although some gender differences were observed. Male school children who were overweight or obese experienced poorer physical functioning, compared to healthy weight males. Obesogenic risk behaviours, except for vegetable consumption, were all significantly associated to HRQoL among males. Female school children reported significantly lower emotional functioning and male school children reported significantly lower school functioning. Overweight and obese school children were more likely to consume soft drinks and takeaway foods, and less likely to meet physical activity recommendations on five days in the previous week.

The negative emotional and social functioning experiences of young people who have overweight/obesity have previously been observed [44, 45]. In particular, overweight and obese young people can experience weight and appearance-related bullying and stigma. It is of interest that overweight/obesity among male school children was associated with poorer HRQoL in this study. Negative social and emotional experiences associated to appearance and weight stigma surrounding thinness is more commonly directed toward females [46]. It is possible that gendered body ideals do not become enforced until adolescence or older and this younger female cohort were yet to be exposed to such ideals [47]. It is also possible that social desirability surrounding health behaviours, which again is disproportionately directed towards females [48], may led to under and over-reporting of health behaviours and functioning. Our previous study demonstrated a relationship between actual body weight, perceived weight status, and HRQoL, however this relationship was sex-specific suggesting the importance of considering socially driven body ideals for males and females [49]. This may have impacted upon results and subsequent significant relationships identified.

The physiological and psychological benefits of physical activity for young school children have been widely reported and this is consistent with current findings [50]. It has been shown among children that there is a dose response relationship between greater physical activity levels, and improved blood cholesterol, blood pressure, bone density and overweight/obesity outcomes [51]. There are also protective effects of physical activity during childhood, including psychological and social opportunities such as building resilience, experiencing teamwork, and goal setting [52]. Such protective effects may support and enable positive functioning, thus improving HRQoL. It is also possible that young children who are experiencing poorer social and emotional functioning may also experience unique barriers in engaging in health behaviours, such as low motivation and lower interest in socialising with peers [53, 54]. The relationship between physical activity and emotional health outcomes has been shown to be bi-directional and it is therefore not possible to draw conclusions on causality in this study.

As previously described, the social environment within which young people interact is rapidly evolving. Indeed it is not overly surprising that 43% of school children in this study exceeded daily screen time recommendations (not reported). Similarly, it was not overly surprising that adhering to screen time recommendations was related to improve HRQoL scores across all domains for males, and for social functioning for females. It has been purported that young people engaging in higher levels of computer, phone and TV use, may displace physical activity (and associated protective benefits) with screens [55]. Similar to physical activity, young people experiencing poorer psychological health may turn to internet-based services for information seeking and support, and bi-directional relationships are probable. It has also been suggested that the connection and relationship experiences on online networking may not offer equivalent protective benefits that can be achieved through face-to-face friendships and networking [56]. Given the substantial time spent using technology daily and the increasing social activities that now occur online, the health effects of screen use are of current and future public health interest. Importantly, it is a consideration that there may be potential detrimental effects if young people experiencing psych-social health concerns are disallowed information seeking and support that might be achieved online [57, 58]. For this reason, and the increasing habitual usage of technology and online social media platforms among young people, further investigation of both the potential negative and positive effects of screen use is warranted.

Fruit consumption has been proposed as an indicator of overall diet quality among young people [59]. It is therefore possible that the positive and consistent relationship between fruit consumption and improved psycho-social health observed in this study, is an indicator of the psychological benefits of an overall healthful dietary pattern. Studies have shown that diets high in fruit, vegetables and wholefoods, and low in ‘extras’ foods such as soft drinks and fast foods, support improved mental and brain health [60–62]. Fruits that are nutrient and antioxidant rich can lower emotional health problems through reducing systematic inflammation, which has been shown to promote poor mental health [59]. In addition, consumption of fruits as part of a healthful diet promote gut microbiota diversity, which recent evidence suggests is a major contributor to emotional health outcomes [63]. Soft drink consumption, which was significantly associated to poorer psycho-social health among male school children, can both increase systematic inflammation and alter gut bacteria, sending negative nerve signals thus impacting upon emotional health [64]. Previous research has indicated that reverse causality does not explain the relationship between diet and mental health [65]. Although study was conducted amongst an adult population and as such it is possible that children experiencing poorer psycho-social health may be less inclined to consume a healthful diet. Given that young people are dependent on family and carer support for the provision of food, it is possible that school children experiences of socio-economic disadvantage or lack of parental support may explain the relationship between fruit consumption and HRQoL.

### Strengths and limitations

This study was strengthened by the opt-out approach which led to large participation rates that have not previously been observed in health behavioural monitoring systems in Australia. It has previously been shown that opt-in approaches significantly underestimates mean BMI-Z scores and prevalence of overweight and obesity compared to opt-out [28]. As such, our study can be considered a closely accurate description of current health status among Victorian regional school children, in comparison to studies employing opt-in procedures. The outcome measure of HRQoL is a validated, has high internal consistency, and has previously been used in school and community-based samples of children [35]. Despite strengths, this study was limited by cross-sectional design and our findings do not provide insight into causality. Behavioural measures were self-reported and it is possible that our findings did not adequately capture the true daily behaviours of Victorian children. As discussed, social desirability may have introduced biases in our results.

### Implications for practice

It is established that improvements in obesogenic risk behaviours are likely to have positive benefits on weight status and long term health. Our study suggests such behaviours may also have significant implications on more immediate quality of life including physical, emotional and social health. This holds important implications for current existing school and community health efforts to curb and prevent obesity through facilitating nutrition and physical activity behaviours as such efforts could be tailored to incorporate both physical and psycho-social health. This study also identifies the current health status of a large, representative sample of Victorian regional school-children. Differences in overweight/obesity prevalence were not significant in terms of language spoken at home and SEIFA indexes of socio-economic disadvantage. However there were indications of a trajectory which inappropriately affected non-English home speaking children (overweight/obesity prevalence=34%) compared to English home speaking children (26%). There are known unique health challenges facing children and families in remote and regional areas, and these have been shown to be most pronounced among CALD communities [66, 67]. Our study warrants the broader monitoring of health risk and protective factors among children within the unique social, economic and culture environments within which they live. Future research is needed to investigate the longitudinal relationships between overweight/obesity, underlying health behaviours, and psycho-social health, in the rapidly evolving typical life of an Australian young person.

## Conclusion

The imperative to intervene in childhood to enable health behaviours is warranted for reasons above and beyond longer term physical health. Our results demonstrate that emotional, social, school, physical functioning are closely connected to the health behaviours which impact upon children’s health later in life. There is clearly great public health and economic potential in aligning overweight/obesity prevention, psycho-social health, and health behavioural promotion efforts. Our study findings demonstrates this alignment is warranted.

## Additional file

Additional file 1: **Table S1.** Mean and standard deviation Health Related Quality of Life scores for males and females. (DOCX 11 kb)

## References

[1] Atay Z, Bereket A (2016). Current status on obesity in childhood and adolescence: Prevalence, etiology, co-morbidities and management. Obesity Medicine.

[2] Chung A, Backholer K, Wong E, Palermo C, Keating C, Peeters A (2016). Trends in child and adolescent obesity prevalence in economically advanced countries according to socioeconomic position: a systematic review. Obesity reviews.

[3] Abarca-Gómez L, Abdeen ZA, Hamid ZA, Abu-Rmeileh NM, Acosta-Cazares B, Acuin C (2017). Worldwide trends in body-mass index, underweight, overweight, and obesity from 1975 to 2016: a pooled analysis of 2416 population-based measurement studies in 128· 9 million children, adolescents, and adults. The Lancet.

[4] Chung A, Peeters A, Gearon E, Backholer K. Contribution of discretionary food and drink consumption to socio-economic inequalities in children’s weight: prospective study of Australian children. Int J Epidemiol. 2018. 10.1093/ije/dyy020 29514246

[5] Sahoo K, Sahoo B, Choudhury AK, Sofi NY, Kumar R, Bhadoria AS (2015). Childhood obesity: causes and consequences. J Family Med Prim Care.

[6] Williams EP, Mesidor M, Winters K, Dubbert PM, Wyatt SB (2015). Overweight and obesity: prevalence, consequences, and causes of a growing public health problem. Curr Obes Rep.

[7] Ludwig DS. Epidemic childhood obesity: Not yet the end of the beginning. Pediatrics. 2018:e20174078. 10.1542/peds.2017-4078 PMC5847089 29483198

[8] Rankin J, Matthews L, Cobley S, Han A, Sanders R, Wiltshire HD, et al. Psychological consequences of childhood obesity: psychiatric comorbidity and prevention. Adolescent health, medicine and therapeutics. 2016;7:125. 10.2147/AHMT.S101631 PMC5115694 27881930

[9] Sanders RH, Han A, Baker JS, Cobley S (2015). Childhood obesity and its physical and psychological co-morbidities: a systematic review of Australian children and adolescents. Eur J Pediatr.

[10] Mühlig Y, Antel J, Föcker M, Hebebrand J (2016). Are bidirectional associations of obesity and depression already apparent in childhood and adolescence as based on high-quality studies? A systematic review. Obes Rev.

[11] Morrison KM, Shin S, Tarnopolsky M, Taylor VH (2015). Association of depression & health related quality of life with body composition in children and youth with obesity. J Affect Disord.

[12] Mansur RB, Brietzke E, McIntyre RS (2015). Is there a “metabolic-mood syndrome”? A review of the relationship between obesity and mood disorders. Neurosci Biobehav Rev.

[13] Viner RM, Ross D, Hardy R, Kuh D, Power C, Johnson A, et al. Life course epidemiology: recognising the importance of adolescence: BMJ Publishing Group Ltd; 2015. 10.1136/jech-2014-205300 PMC4515995 25646208

[14] Simmonds M, Llewellyn A, Owen C, Woolacott N (2016). Predicting adult obesity from childhood obesity: a systematic review and meta-analysis. Obes Rev.

[15] Karimi M, Brazier J (2016). Health, health-related quality of life, and quality of life: what is the difference?. PharmacoEconomics.

[16] Rajmil L, Herdman M, de Sanmamed M-JF, Detmar S, Bruil J, Ravens-Sieberer U (2004). Generic health-related quality of life instruments in children and adolescents: a qualitative analysis of content. J Adolesc Health.

[17] Jacka FN, Cherbuin N, Anstey KJ, Sachdev P, Butterworth P (2015). Western diet is associated with a smaller hippocampus: a longitudinal investigation. BMC Med.

[18] Swallen KC, Reither EN, Haas SA, Meier AM (2005). Overweight, obesity, and health-related quality of life among adolescents: the National Longitudinal Study of Adolescent Health. Pediatrics.

[19] Wake M, Clifford S, Patton G, Waters E, Williams J, Canterford L (2013). Morbidity patterns among the underweight, overweight and obese between 2 and 18 years: population-based cross-sectional analyses. Int J Obes (Lond).

[20] Boyd D (2014). It's complicated: The social lives of networked teens: Yale University Press.

[21] Wolnicka K, Jarosz M, Jaczewska-Schuetz J, Taraszewska AM. Differences in the prevalence of overweight, obesity and underweight among children from primary schools in rural and urban areas. Ann Agric Environ Med. 2016;23(2). 10.5604/12321966.1203902 27294644

[22] Bolton K, Kremer P, Rossthorn N, Moodie M, Gibbs L, Waters E (2014). The effect of gender and age on the association between weight status and health-related quality of life in Australian adolescents. BMC Public Health.

[23] Jia H, Lubetkin EI (2005). The impact of obesity on health-related quality-of-life in the general adult US population. J Public Health.

[24] Andersen JR, Aasprang A, Karlsen T-I, Natvig GK, Våge V, Kolotkin RL (2015). Health-related quality of life after bariatric surgery: a systematic review of prospective long-term studies. Surg Obes Relat Dis.

[25] Chen G, Ratcliffe J, Olds T, Magarey A, Jones M, Leslie E (2014). BMI, health behaviors, and quality of life in children and adolescents: a school-based study. Pediatrics.

[26] Dumuid D, Olds T, Lewis LK, Martin-Fernández JA, Katzmarzyk PT, Barreira T, et al. Health-related quality of life and lifestyle behavior clusters in school-aged children from 12 countries. The Journal of pediatrics. 2017;183:178-83. e2. 10.1016/j.jpeds.2016.12.048 28081885

[27] Tigges BB (2003). Parental consent and adolescent risk behavior research. J Nurs Scholarsh.

[28] Strugnell C, Orellana L, Hayward J, Millar L, Swinburn B, Allender S. Active (Opt-In) Consent Underestimates Mean BMI-z and the Prevalence of Overweight and Obesity Compared to Passive (Opt-Out) Consent. Evidence from the Healthy Together Victoria and Childhood Obesity Study. Int J Environ Res Public health. 2018;15(4):747. 10.3390/ijerph15040747 PMC5923789 29652831

[29] Crooks N, Strugnell C, Bell C, Allender S (2016). A sustainable and high participation childhood obesity monitoring system in regional Victoria, Australia. Australian and New Zealand Obesity Society annual meeting.

[30] Card A, Manske S, Mammen G, King M, Gleddie D, Schwartz M (2012). Core Indicators and Measures of Youth Health Physical Activity & Sedentary Behaviour Module: Indicators and Questions to use with Youth Respondents and/or School Setting Assessment.

[31] Wong SL, Leatherdale ST, Manske SR (2006). Reliability and validity of a school-based physical activity questionnaire. Med Sci Sports Exerc.

[32] Parletta N, Frensham L, Peters J, O'Dea K, Itsiopoulos C. Validation of a Simple Dietary Questionnaire with adolescents in an Australian population. under review. 2013.

[33] Varni JW, Limbers CA, Burwinkle TM. How young can children reliably and validly self-report their health-related quality of life?: an analysis of 8,591 children across age subgroups with the PedsQL 4.0 Generic Core Scales. Health And Quality Of Life Outcomes. 2007;5:1-. 10.1186/1477-7525-5-1 PMC1769360 17201920

[34] Varni JW, Seid M, Rode CA. The PedsQL™: measurement model for the pediatric quality of life inventory. Medical Care. 1999:126–39. 10.1097/00005650-199902000-00003 10024117

[35] Varni JW, Seid M, Kurtin PS. PedsQL™ 4.0: Reliability and validity of the Pediatric Quality of Life Inventory™ Version 4.0 Generic Core Scales in healthy and patient populations. Medical Care. 2001:800–12. 10.1097/00005650-200108000-00006 11468499

[36] Commonwealth Department of Health. Australia's Physical Activity and Sedentary Behaviour Guidelines for Children (5-12 years). Canberra2014.

[37] LeBourgeois MK, Giannotti F, Cortesi F, Wolfson AR, Harsh J (2005). The relationship between reported sleep quality and sleep hygiene in Italian and American adolescents. Pediatrics.

[38] Shahid A, Wilkinson K, Marcu S, Shapiro CM (2011). School sleep habits survey.

[39] Shahid A, Wilkinson K, Marcu S, Shapiro C. Children’s Sleep Habits Questionnaire (CSHQ) in Stop. That and One hundred other sleep scales. 2012:119–22.

[40] Shahid A, Wilkinson K, Marcu S, Shapiro CM. Pittsburgh Sleep Quality Index (PSQI). Stop, that and one hundred other sleep scales. Springer. 2011:279–83.

[41] Cole TJ, Bellizzi MC, Flegal KM, Dietz WH (2000). Establishing a standard definition for child overweight and obesity worldwide: international survey. Bmj.

[42] Health N (2013). Council MR.

[43] Pink B (2011). Census of Population and Housing: Socio-economic Indexes for Areas (SEIFA). Australia. Australia: Australian Bureau of Statistics.

[44] Young-Hyman D, Tanofsky-Kraff M, Yanovski SZ, Keil M, Cohen ML, Peyrot M (2006). Psychological status and weight-related distress in overweight or at-risk-for-overweight children. Obesity (Silver Spring).

[45] Hayden-Wade HA, Stein RI, Ghaderi A, Saelens BE, Zabinski MF, Wilfley DE (2005). Prevalence, characteristics, and correlates of teasing experiences among overweight children vs. non-overweight peers. Obes Res.

[46] Lawler M, Nixon E (2011). Body dissatisfaction among adolescent boys and girls: the effects of body mass, peer appearance culture and internalization of appearance ideals. J Youth Adolescence.

[47] Smolak L (2004). Body image in children and adolescents: where do we go from here?. Body image.

[48] Van de Mortel TF. Faking it: social desirability response bias in self-report research. Australian Journal of Advanced Nursing, The. 2008;25(4):40.

[49] Hayward J, Millar L, Petersen S, Swinburn B, Lewis AJ (2014). When ignorance is bliss: weight perception, body mass index and quality of life in adolescents. Int J Obes (Lond).

[50] Biddle SJ, Asare M. Physical activity and mental health in children and adolescents: a review of reviews. Brit J Sports Med. 2011:bjsports90185. 10.1136/bjsports-2011-090185 21807669

[51] Janssen I, LeBlanc AG (2010). Systematic review of the health benefits of physical activity and fitness in school-aged children and youth. Int J Behav Nutr Phys Act.

[52] Strauss RS, Rodzilsky D, Burack G, Colin M (2001). Psychosocial correlates of physical activity in healthy children. Arch Pediatr Adolesc Med.

[53] Lewis BA, Marcus BH, Pate RR, Dunn AL (2002). Psychosocial mediators of physical activity behavior among adults and children. Am J Prev Med.

[54] O'Dea JA (2003). Why do kids eat healthful food? Perceived benefits of and barriers to healthful eating and physical activity among children and adolescents. J Am Diet Assoc.

[55] Tremblay MS, LeBlanc AG, Kho ME, Saunders TJ, Larouche R, Colley RC (2011). Systematic review of sedentary behaviour and health indicators in school-aged children and youth. Int Behav Nutr Phys Activ.

[56] Desjarlais M, Joseph JJ (2017). Socially interactive and passive technologies enhance friendship quality: An investigation of the mediating roles of online and offline self-disclosure. Cyberpsychol Behav Soc Netw.

[57] Hoare E, Milton K, Foster C, Allender S (2017). Depression, psychological distress and Internet use among community-based Australian adolescents: a cross-sectional study. BMC Public Health.

[58] Boberska M, Szczuka Z, Kruk M, Knoll N, Keller J, Hohl DH (2018). Sedentary behaviours and health-related quality of life. A systematic review and meta-analysis. Health Psychol Rev.

[59] Jacka FN, Kremer PJ, Berk M, de Silva-Sanigorski AM, Moodie M, Leslie ER (2011). A prospective study of diet quality and mental health in adolescents. PloS One.

[60] Li Y, Lv M-R, Wei Y-J, Sun L, Zhang J-X, Zhang H-G (2017). Dietary patterns and depression risk: a meta-analysis. Psychiatry Res.

[61] Psaltopoulou T, Sergentanis TN, Panagiotakos DB, Sergentanis IN, Kosti R, Scarmeas N (2013). Mediterranean diet, stroke, cognitive impairment, and depression: a meta-analysis. Ann Neurol.

[62] O’neil A, Quirk SE, Housden S, Brennan SL, Williams LJ, Pasco JA (2014). Relationship between diet and mental health in children and adolescents: a systematic review. Am J Public Health.

[63] Montiel-Castro AJ, González-Cervantes RM, Bravo-Ruiseco G, Pacheco-López G (2013). The microbiota-gut-brain axis: neurobehavioral correlates, health and sociality. Front Integr Neurosci.

[64] Rodriguez-Castaño GP, Caro-Quintero A, Reyes A, Lizcano F (2017). Advances in gut microbiome research, opening new strategies to cope with a western lifestyle. Front Gen.

[65] Jacka FN, Cherbuin N, Anstey KJ, Butterworth P (2015). Does reverse causality explain the relationship between diet and depression?. J Affect Disord.

[66] Swinburn BA, Sacks G, Hall KD, McPherson K, Finegood DT, Moodie ML (2011). The global obesity pandemic: shaped by global drivers and local environments. The Lancet.

[67] Armstrong BK, Gillespie JA, Leeder SR, Rubin GL, Russell LM (2007). Challenges in health and health care for Australia. Med J Aust.

