# Supplementary material for: Associations between combined overweight and obesity, lifestyle behavioural risk and quality of life among Australian regional school children: baseline findings of the Goulburn Valley health behaviours monitoring study
Source: Health Qual Life Outcomes. 2019 Jan 18;17:16. doi: 10.1186/s12955-019-1086-0 (PMC6339321; doi:10.1186/s12955-019-1086-0)
Supplement: Supplementary file 1 — Table S1. Mean and standard deviation Health Related Quality of Life scores for males and females. (DOCX 11 kb) [file 12955_2019_1086_MOESM1_ESM.docx]

**Supplementary File 1.** Mean and standard deviation Health Related Quality of Life scores for males and females

| **HRQoL** | **Males** | **Females** | **P value** |
| --- | --- | --- | --- |
| Physical m(95%CI) | 84.4 (83.0, 85.9) | 83.7 (82.3, 85.2) | NS |
| Emotional m(95%CI) | **74.0 (72.2, 75.8)** | **68.2 (66.0, 70.3)** | **P<0.05** |
| Social m(95%CI) | 78.0 (75.9, 80.0) | 79.0 (76.9, 81.0) | NS |
| School m(95%CI) | **73.1 (71.3, 74.8)** | **77.1 (75.4, 78.9)** | **P<0.05** |
| Global m(95%CI) | 78.2 (76.9, 79.6) | 77.9 (76.4, 79.4) | NS |
